# Natural and synthetic cathelicidin peptides with anti-microbial and anti-biofilm activity against *Staphylococcus aureus*

**DOI:** 10.1186/1471-2180-11-114

**Published:** 2011-05-23

**Authors:** Scott N Dean, Barney M Bishop, Monique L van Hoek

**Affiliations:** 1 Department of Biology, George Mason University, Manassas, VA, 20110, USA; 2 Department of Chemistry and Biochemistry, George Mason University, Manassas, VA, 20110, USA; 3 Department of Molecular and Microbiology and National Center for Biodefense and Infectious Diseases, George Mason University, Manassas, VA, 20110, USA

## Abstract

**Background:**

Chronic, infected wounds typically contain multiple genera of bacteria, including *Staphylococcus aureus*, many of which are strong biofilm formers. Bacterial biofilms are thought to be a direct impediment to wound healing. New therapies that focus on a biofilm approach may improve the recovery and healing rate for infected wounds. In this study, cathelicidins and related short, synthetic peptides were tested for their anti-microbial effectiveness as well as their ability to inhibit the ability of *S. aureus *to form biofilms.

**Results:**

The helical human cathelicidin LL-37 was tested against *S. aureus*, and was found to exhibit effective anti-microbial, anti-attachment as well as anti-biofilm activity at concentrations in the low μg/ml range. The effect of peptide chirality and associated protease-resistance was explored through the use of an all-D amino acid peptide, D-LL-37, and in turn compared to scrambled LL-37. Helical cathelicidins have been identified in other animals such as the Chinese cobra, *Naja atra *(NA-CATH). We previously identified an 11-residue imperfectly repeated pattern (ATRA motif) within the sequence of NA-CATH. A series of short peptides (ATRA-1, -2, -1A), as well as a synthetic peptide, NA-CATH:ATRA1-ATRA1, were designed to explore the significance of the conserved residues within the ATRA motif for anti-microbial activity. The CD spectrum of NA-CATH and NA-CATH:ATRA1-ATRA1 revealed the structural properties of these peptides and suggested that helicity may factor into their anti-microbial and anti-biofilm activities.

**Conclusions:**

The NA-CATH:ATRA1-ATRA1 peptide inhibits the production of biofilm by *S. aureus *in the presence of salt, exhibiting anti-biofilm activity at lower peptide concentrations than NA-CATH, LL-37 and D-LL-37; and demonstrates low cytoxicity against host cells but does not affect bacterial attachment. The peptides utilized in this anti-biofilm approach may provide templates for a new group of anti-microbials and potential future topical therapeutics for treating chronic wound infections.

## Background

Staphylococci are common commensal bacteria of the skin [1], as well as important pathogens in foreign-body infections [2]. The gram-positive *Staphylococcus (S.) aureus *is a major human pathogen. It is the cause of many nosocomial infections, including life-threatening diseases such as toxic shock syndrome, sepsis and endocarditis [3]. *S. aureus *infections account for approximately 19,000 deaths per year in the United States [4]. The emergence of multi-drug resistant strains of *S. aureus*, such as methicillin-resistant *S. aureus *(MRSA), has intensified the need for new treatments [5]. The danger of untreatable staphylococcal infections highlights the importance of new anti-microbial drug discovery.

It has been discovered that chronic, infected wounds are often infected with strong biofilm forming bacteria, such as *S. aureus *[6], and it is now thought that the presence of biofilm actively prevents the healing of these wounds [7]. Chronic wounds can arise as a result of pressure sores, venous leg ulcers, diabetic foot ulcers or combat wounds, for example. While physical debridement can assist the healing of these wounds, biofilm-focused therapeutic approaches can promote more rapid healing in a large percent of patients [7]. This biofilm-centric philosophy may represent a modern strategy to treat chronic, infected wounds in which reducing the ability of the bacteria to form biofilm is itself the critical goal. In this strategy, subsequent healing by the body or treatment with antibiotics is then more effective. In support of this approach to wound treatment, we tested natural and novel peptides for their anti-microbial anti-attachment as well as anti-biofilm effectiveness.

Anti-microbial peptides (AMPs) are essential components of innate immunity in humans and other higher organisms, contributing to our first line of defense against infection [8]. Despite co-evolution with bacteria, AMPs have retained their advantage and bacteria have yet to develop wide-spread resistance. Accordingly, there is growing interest in the therapeutic application of these molecules. Their amino acid sequences, net-positive charge, amphipathicity, and very small size allow AMPs to bind to and disrupt membranes of microbes [9]. Other research has shown that AMPs can also inhibit cell wall, nucleic acid, and protein biosynthesis [10]. AMPs have immunomodulatory effects as well: they are chemotactic for many leukocytes, drawing them to the site of infection or inflammation. They have also been shown to be capable of binding and neutralizing lipopolysaccharides, promoting angiogenesis and wound healing, and exerting anti-tumor activity [11]. There are only a few examples of peptides with anti-biofilm activity against *S. aureus*. Synthetic peptide mimics of the ceragenin class [12-14] and an RNAIII-inhibiting peptide [15] have been shown to reduce *S. aureus *biofilm formation.

The cathelicidin family of AMPs is a large and diverse group of peptides that range from 12-80 amino acid residues in length. Cathelicidins are identified based on a conserved N-terminal domain, the cathelin domain, present in the inactive precursor peptide [16]. These can be found in their precursor form in the granules of natural killer T cells, neutrophils, and in the mucosal epithelia of the lungs, with the functional anti-microbial cathelicidin peptide generated through proteolytic removal of the cathelin domain as part of the secretion process [17]. The sequence diversity of cathelicidins translates into the peptides demonstrating structural diversity, and the peptides can be grouped into sub-classes based on shared structural features. The helical cathelicidins, the largest of the cathelicidin structural classes, adopt a helical conformation when interacting with membranes by folding to make amphipathic alpha-helices. The knowledge of cathelicidin structural and functional properties is largely based on observations from the highly studied human cathelicidin, LL-37 [18].

LL-37 is derived from the C-terminus of the human CAP-18 protein. It is a 37 residue cationic peptide which forms an alpha-helix when in contact with bacterial membranes or sodium dodecyl sulfate (SDS). This peptide has broad-spectrum anti-microbial activity against gram-negative and gram-positive bacteria, including reported effectiveness against *S. aureus *(EC50 = 1.6 μg/ml) [19]. Another group of peptides, the human β-defensins, have been tested against this species. However, β-defensins were deemed mostly ineffective [20]. Two other cathelicidin peptides, the bovine and porcine myeloid anti-microbial peptides, demonstrated effective anti-microbial activity when tested against *S. aureus *[21].

MRSA strains appear to be less sensitive to LL-37 [22], demonstrating the need to identify more effective AMPs. We synthesized a peptide mimetic of LL-37, a synthetic D-LL-37 peptide, in which every amino acid was changed to the D-form (the enantiomer). Peptides in the D-amino acid form are resistant to proteases such as trypsin [23], which may be present in wound exudate. If chirality is not important for its anti-microbial properties, this could potentially be an effective and protease-resistant AMP. Using this peptide, we examined the role of chirality in LL-37's effectiveness against *S. aureus*.

A recently identified helical cathelicidin from the elapid snake *Bungarus fasciatus *(BF) was found to be effective against *S. aureus *(minimum inhibitory concentration (MIC) of 4.7 μg/ml) [21]. A related cathelicidin has been discovered in the elapid snake *Naja atra*, the Chinese Cobra, but it has not been tested against *S. aureus*. We previously observed that the *Naja atra *cathelicidin (NA-CATH) contains an imperfect, repeated 11 amino acid motif (ATRA), larger than had been previously described by Zhao *et al. *[24-26], and that small peptides based on this motif displayed antimicrobial activity. We designed and synthesized a version of NA-CATH with a perfect repeat (NA-CATH:ATRA1-ATRA1) in order to explore the significance of the conserved residues within the ATRA motif and how they impacted anti-microbial activity. The CD spectra of NA-CATH and NA-CATH:ATRA1-ATRA1 were obtained to examine the role of helicity in anti-microbial and anti-biofilm activity. Thus, we have developed two synthetic peptides, D-LL-37 and NA-CATH:ATRA1-ATRA1, both of which have significant anti-microbial and anti-biofilm activity against *S. aureus*. The D-LL-37 peptide represents a protease-resistant enantiomer of the natural human cathelicidin, while NA-CATH:ATRA1-ATRA1 is an improvement to a natural snake cathelicidin. We envision that such novel, synthetic, broad-spectrum peptides could be incorporated into a topical wound treatment or dressing.

## Results

### 2. Results

#### 2.1 Anti-microbial performance

##### a. LL-37 and NA-CATH are anti-microbial against *S. aureus*

**Figure 1:**
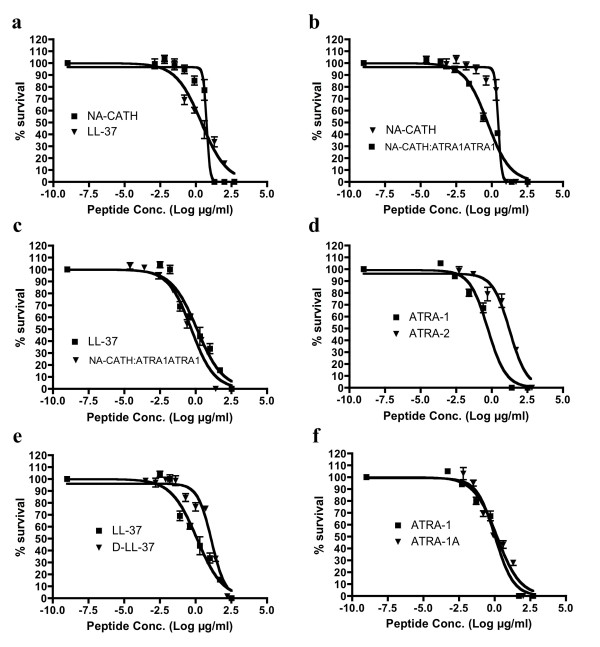
**Effectiveness of anti-microbial peptides against *S. aureus***. Percent (%) survival was calculated by counting CFUs, after 3 hr incubations with various peptide concentrations in 10 mM sodium phosphate buffer (pH 7.4). The EC50 is reported. **a**, The EC50s were found to be 2.9 μg/ml for NA-CATH and 1.3 μg/ml for LL-37. **b**, EC50s were found to be 0.51 μg/ml for NA-CATH:ATRA1-ATRA1 and 2.9 μg/ml for NA-CATH. **c**, EC50s were found to be 0.51 μg/ml for NA-CATH:ATRA1-ATRA1 and 1.3 μg/ml for LL-37. **d**, EC50s were found to be 0.52 μg/ml for ATRA-1 and 18 μg/ml for ATRA-2. **e**, EC50s were found to be 13 μg/ml for D-LL-37 and 1.3 μg/ml for LL-37. **f**, EC50s were found to be 0.73 μg/ml for ATRA-1A and 0.52 μg/ml for ATRA-1. Curves were fit to the data, and R^2 ^values were as follows: 0.97 for NA-CATH:ATRA1-ATRA1; 0.98 for NA-CATH; 0.95 for LL-37; 0.95 for D-LL-37; 0.98 for ATRA-1; 0.96 for ATRA-2; 0.96 for ATRA-1A.

The peptide sequences are described in Table 1. The anti-microbial effectiveness of NA-CATH was tested against *S. aureus*, and the performance of this peptide was compared to the activity of the well-studied cathelicidin LL-37. The EC50 for NA-CATH was found to be 2.9 μg/ml (Figure 1a). The peptide NA-CATH:ATRA1-ATRA1 incorporates modification to NA-CATH in which the second ATRA motif has been changed to match the sequence of the first ATRA motif (Table 2). This synthetic cathelicidin had an EC50 value that was determined to be 0.51 μg/ml, more effective against *S. aureus *(p < 0.05) than the parental NA-CATH (Figure 1b), but not statistically different from LL-37 (Figure 1c). In agreement with reported potencies [19], we found that the EC50 for LL-37 is 1.27 μg/ml. This is similar to the level of LL-37 reported in human plasma (1.18 μg/ml) [27], suggesting that this is a physiologically relevant potency of LL-37.

**Table 1:** Peptides used in this study

Antimicrobial Peptides	Sequence	Net charge
**NA-CATH**	**KRFKKFFKKLK**NSVK**KRAKKFFKKPK**VIGVTFPF	15
**NA-CATH-ATRA1-ATRA1**	**KRFKKFFKKLK**NSVK**KRFKKFFKKLK**VIGVTFPF	15
**ATRA-1**	KRFKKFFKKLK-NH2	8
**ATRA-2**	KRAKKFFKKPK-NH2	8
**ATRA-1A**	KRAKKFFKKLK-NH2	8
**LL-37**	LLGDFFRKSKEKIGKEFKRIVQRIKDFLRNLVPRTES	6
**D-LL-37**	*LLGDFFRKSKEKIGKEFKRIVQRIKDFLRNLVPRTES*	6
**Scrambled LL-37**	GLKLRFEFSKIKGEFLKTPEVRFRDIKLKDNRISVQR	6

This table indicates the Sequence and charges of the antimicrobial peptides used. The ATRA motif is indicated in BOLD. The 3d and 10th positions of the ATRA peptides are underscored. The D-amino acids are indicated in italics.

**Table 2:** EC50s of AMPs against *S. aureus*

Antimicrobial Peptides	Molecular weight (g/mol)	EC50 (μg/ml)	95% CI	EC50 (μM)
**NA-CATH**	5885.50	2.85	1.22-6.69	0.48
**NA-CATH-ATRA1-ATRA1**	5977.60	0.51	0.25-1.01	0.09
**ATRA-1**	2409.06	0.52	0.25-1.11	0.22
**ATRA-2**	2316.96	18.0	7.67-41.8	7.77
**ATRA-1A**	2332.96	0.73	0.33-1.62	0.31
**LL-37**	5177.42	1.27	0.44-3.72	0.25
**D-LL-37**	5177.42	12.7	6.48-24.9	2.45

This table indicates the EC50 of the peptides against *S. aureus *in an anti-microbial assay. (*) The molecular weight reported here for each peptide reflects the TFA salts of the peptides. These molecular weights were then used to convert the EC50 in μg/ml to μM, to enable comparisons on a molecule by molecule basis.

##### b. Synthetic peptides demonstrate anti-microbial activity against *S. aureus*

*S. aureus *was also subjected to treatment with four synthetic peptides (Table 1), ATRA-1, ATRA-2, ATRA-1A, and NA-CATH:ATRA1-ATRA1, which represent variations on the ATRA-repeated motif of NA-CATH. The two ATRA peptides, ATRA-1 and ATRA-2, differ by two residues at the 3rd (F/A) and 10th (L/P) position. This has been shown to affect the anti-microbial activity of those peptides against *Francisella novicida, Escherichia coli *[25] and *Aggregatibacter actinomycetemcomitans *[26]. The EC50 values of ATRA-1 and ATRA-2 were determined to be statistically different (p < 0.05) at 0.52 and 18 μg/ml, respectively (Table 2), with non-overlapping 95% Confidence Intervals (Figure 1d). These two peptides have the same net charge of +8, highly similar sequence and the same length of 11 amino acid residues. The ATRA-1A peptide is a variation on the ATRA-1 peptide. ATRA-1A differs from the ATRA-1 peptide in the 3^rd ^position, which in our previous studies with gram-negative bacteria improved its anti-microbial activity. The EC50 against *S. aureus *of ATRA-1A was found to be 0.73 μg/ml (Figure 1f); the additional alanine did not significantly improve its activity, as the EC50 for ATRA-1 was determined as 0.52 μg/ml (Table 2), with overlapping confidence intervals.

When examined on a molar basis (Table 2), taking into account the activity per molecule of peptide, whether short or long, it can be seen that the short, synthetic ATRA-1A peptide is as potent as the full-length NA-CATH against *S. aureus *(Figure 1a, b). It can also be seen that LL-37 is still a more effective anti-microbial peptide than either of those peptides (Figure 1a). However, altering the NA-CATH peptide to have a perfect ATRA repeat (NA-CATH:ATRA1-ATRA1) generated the most potent peptide of all, judged either in terms of molarity or μg/ml (Figure 1b, c).

##### c. Effect of Chirality: D- vs L-LL-37 against *S. aureus*

A common concern against the use of anti-microbial peptides as a therapeutic is their potential sensitivity to host or bacterial proteases [28]. In order to generate a protease-resistant peptide mimetic of the human cathelicidin [23], we tested an all-D-amino acid version of LL-37. This peptide is the chiral opposite peptide to LL-37, but has an otherwise identical sequence and net charge. The antimicrobial EC50 value of the D-peptide against *S. aureus *was determined to be 12.7 μg/ml, compared to 1.27 μg/ml for wild-type LL-37 (Table 2, Figure 1e). The apparently decreased potency of D-LL-37 may reflect deficiencies in the ability of the peptide isomer to interact effectively with the gram-positive bacterial cell membrane, or it may have diminished helical character relative to the L-isomer, though this is not reported in the literature. Alternatively, it may indicate the existence of a heretofore unidentified chiral binding target for the LL-37 peptide in *S. aureus*.

#### 2.2 Hemolytic activity of peptides

The hemolytic activity of each of the peptides was determined using 2% horse erythrocytes as previously described [29]. In these assays, no significant hemolysis was demonstrated by any of the tested peptides up to a concentration of 100 μg/ml (data not shown). We previously reported low hemolytic activity of the ATRA series of peptides [26]. At 100 μg/ml, NA-CATH:ATRA1-ATRA1 did not elicit statistically significant hemolysis compared to PBS (Fisher Scientific) (pH 7) or to the parent compound, NA-CATH (p = 0.98). Other studies have examined hemolytic activity of cathelicidins up to 200 μg/ml, and found similarly low levels in full-length LL-37 and short ATRA fragments [24,26]. At 100 μg/ml, D-LL-37 also elicited no significant hemolysis and was not statistically significantly different than the L-form (p = 0.29 compared to LL-37).

#### 2.3 Inhibition of biofilm formation at sub-anti-microbial concentrations

Another common concern of the utility of antimicrobial peptides as potential therapeutics is the sensitivity of the antimicrobial activity to salt. Multiple studies have shown that LL-37 demonstrates reduced antimicrobial action in environments with high ionic concentrations [30,31] such as in physiologic salt concentration (123-150 mM NaCl). However, LL-37 can inhibit biofilm formation by *P. aeruginosa *[32], *S. epidermidis *[33] and *F. novicida *[25] in media with a high concentrations of salt. In conclusion, although the LL-37 peptide loses its anti-microbial activity in high salt, it retains its anti-biofilm activity.

**Figure 2:**
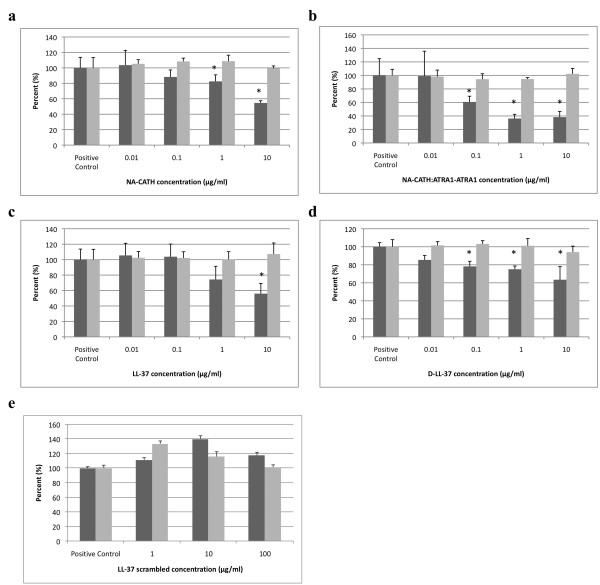
**Anti-biofilm activity of peptides**. Inhibition of *S. aureus *biofilm formation was demonstrated for each of the following peptides. **A**. NA-CATH. **B**. NA-CATH:ATRA1-ATRA1. **C**. LL-37. **D**. D-LL-37. **E**. Scrambled LL-37. Growth (absorbance at 600 nm) is indicated by gray bars with "0 peptide" control set to 100%. Biofilm detection on a polystyrene 96-well plate at 37°C after 24 h of growth in TSB was detected as the absorbance of crystal violet stain (570 nm). Percent biofilm production is indicated by black bars (n = 6), relative to "0 peptide" control. Each experiment is a representative of at least two independent trials. Error bars indicate the standard deviation from the mean. The asterisk (*) indicates statistically different than the positive control (p < 0.01).

In this study, we demonstrate similar salt-independent anti-biofilm activity for NA-CATH, NA-CATH:ATRA1-ATRA1 and D-LL-37 peptides. We incubated various concentrations of NA-CATH, NA-CATH:ATRA1-ATRA1, LL-37, D-LL-37, and scrambled LL-37 with *S. aureus *in biofilm experiments in sterile TSB (relatively high salt) for 24 h. Figure 2 (2a, b, c, d and 2e) shows that levels of bacterial growth (OD600 at 24 hours) were not decreased even at the peptide concentrations equal to that of its calculated EC50 in sterile 10 mM sodium phosphate. The MIC of LL-37 against *S. aureus *was determined to be >400 μg/ml, in TSB (data not shown). When the biofilm production was determined in the presence of varying amounts of peptide, significant inhibition of biofilm formation by each of the peptides (except the scrambled LL-37) was observed at concentrations in which no anti-microbial activity is observed. Thus, wild-type NA-CATH was found to inhibit biofilm formation up to ~50% of control at 10 μg/ml (Figure 2a). NA-CATH:ATRA1-ATRA1 was found to be the most active anti-biofilm peptide, with maximal biofilm inhibition observed at 1 μg/ml, inhibiting ~60% of biofilm formation (Figure 2b).

For LL-37, significant anti-biofilm inhibition for *S. aureus *was observed at 10 μg/ml, inhibiting ~40% biofilm formation (Figure 2c). The anti-biofilm activity of D-LL-37 was very similar to that of LL-37, showing ~40% inhibition at 10 μg/ml (Figure 2d). In other experiments, D-LL-37 at 26 μg/ml was able to inhibit as much as ~80% of the biofilm formation (data not shown). This strong anti-biofilm effect of D-LL-37 was surprising, as it was categorized as an ineffective AMP (Table 2), and was 10 fold less effective than LL-37. This result suggests that anti-microbial activity and anti-biofilm activity of peptides may be due to different mechanisms. For example, the anti-microbial activity could be direct physical interaction of the peptide on the bacterial membrane, while anti-biofilm could be mediated by alteration of bacterial gene expression [32].

The scrambled version of LL-37, having the same charge and net amino-acid composition as LL-37, but lacking significant helical character, showed no inhibition of biofilm formation at any concentration tested (Figure 2e), thus demonstrating sequence specificity of the anti-biofilm effect.

#### 2.4 D- and L-LL-37 effect *S. aureus *biofilm attachment

**Figure 3:**
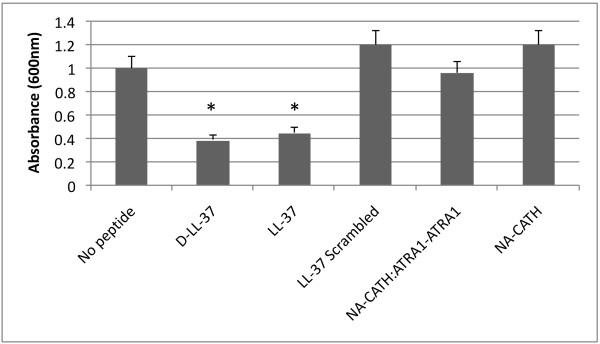
**Attachment assay of *S. aureus *in the presence of peptide**. We tested scrambled LL-37 (negative control), LL-37, D-LL-37, NA-CATH, and NA-CATH:ATRA1-ATRA1 against *S. aureus *(1 h, 37°C) at 1 μg/ml, only allowing for the initial adherence to the wells. For LL-37 and D-LL-37, the measured attachment to the polypropylene wells was significantly decreased (P < 0.01, Student's t test). Scrambled LL-37, NA-CATH, and NA-CATH:ATRA1-ATRA1 did not decrease *S. aureus *adherence.

The attachment of *Staphylococcus spp*. to solid surfaces is largely seen as an essential step in the formation of biofilm. Since most of the peptides tested in our biofilm assays were capable of inhibiting biofilm formation (except for scrambled LL-37), we investigated a possible mechanism for this action. We incubated scrambled LL-37 (negative control), LL-37, D-LL-37, NA-CATH, and NA-CATH:ATRA1-ATRA1 peptides with *S. aureus *in a 1 hr attachment assay at peptide concentrations of 1 ug/ml, examining for the initial adherence to the wells of the 96 well tissue-culture treated plate [32]. For LL-37 and D-LL-37, the measured attachment to the polystyrene wells was significantly decreased (P < 0.01, Student's t test) (Figure 3). Scrambled LL-37, NA-CATH, and NA-CATH:ATRA1-ATRA1 did not decrease *S. aureus *adherence. Thus, both D- and L-forms of the LL-37 peptide were equally effective at inhibiting attachment, which may contribute to their inhibition of biofilm formation. However, the most effective anti-biofilm peptide, NA-CATH:ATRA1-ATRA1 did not inhibit attachment, suggesting that this peptide inhibits biofilm formation through a different mechanism.

#### 2.5 CD Spectral analysis of peptides

**Figure 4:**
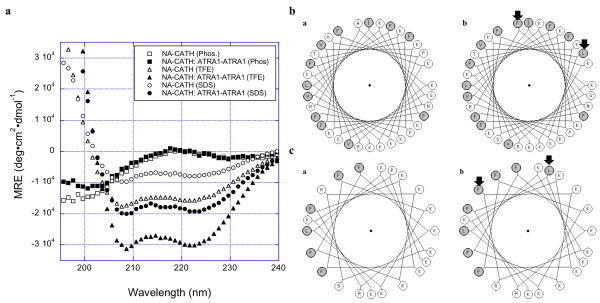
**Circular Dichroism Spectra of NA-CATH and NA-CATH:ATRA1-ATRA1**. Pronounced dichroic minima at 222 and 208 nm are traits of helical peptides. While NA-CATH and NA-CATH:ATRA1-ATRA1 do not show significant helical character in 10 mM sodium phosphate, both peptides exhibit helical structure in 60 mM SDS in 10 mM phosphate buffer (pH 7) and in 50% TFE in 10 mM phosphate buffer (pH 7). Under both conditions, NA-CATH:ATRA1-ATRA1 displayed more pronounced helical character than NA-CATH. **B. Helical Wheel projection**. Helical wheel projections were made with http://kael.org/helical.htm (accessed on 12/15/10). The sequences of (a) NA-CATH and (b) NA-CATH:ATRA1-ATRA1 were projected onto the helical backbone. Altered residues are indicated by the arrows. Shaded residues indicate hypdrophobic residues. **C. ATRA2 vs ATRA1 motifs in helical wheel projection**. To enable easier viewing of contribution of the key differences between the ATRA2 (a) and the ATRA1 (b) motifs to the hydrophobic face of the peptide, each motif is projected alone on the helical wheel in this view. Altered residues are indicated by the arrows. Shaded residues indicate hypdrophobic residues.

Circular dichroism (CD) spectra of the peptides were obtained. Pronounced dichroic minima at 222 and 208 nm are traits of helical peptides (Figure 4a). Cathelcidins often exhibit little helical behavior in aqueous buffer, assuming their helical structure only in association with a biological membrane, a membrane mimic such as SDS, or trifluoroethanol (TFE), a strongly helix-promoting environment. SDS is used to mimic the anionic bacterial membrane [34], and structural studies using this method have provided insight into peptide-membrane interactions. In a previous study, we demonstrated that the ATRA-1 peptide exhibits very strong helical properties, while ATRA-2 peptide had poor helical properties [25,26], probably due to the proline at the 10^th ^position. ATRA-1 was also predicted to present a more cohesive hydrophobic face than ATRA-2 (see below). These characteristics, taken together, may account for the high level of anti-microbial effectiveness displayed by ATRA-1. We hypothesized that compared to the parental NA-CATH (containing both ATRA-1 and ATRA-2 segments), the NA-CATH:ATRA1-ATRA1 peptide may benefit from greater and more stable helical character when interacting with bacterial membranes and that this may contribute to its increased anti-microbial activity [35].

Neither NA-CATH nor NA-CATH:ATRA1-ATRA1 show well-defined secondary structure in 10 mM sodium phosphate (pH 7) (Figure 4A), as expected. However, both peptides appear to adopt a helical conformation in 50% TFE, with the NA-CATH:ATRA1-ATRA1 spectrum indicating significantly more helical character than is noted for the NA-CATH parental peptide. SDS may more closely approximate the conditions associated with the interaction between CAMPs and bacterial membranes, thus CD spectra were also collected for NA-CATH and NA-CATH:ATRA1-ATRA1 in the presence of 60 mM SDS. Both peptides demonstrated helical character under these conditions, but less than they presented in 50% TFE. Again, NA-CATH:ATRA1-ATRA1 demonstrated more helical character than did the wild-type peptide. Moreover, the CD spectrum of NA-CATH:ATRA1-ATRA1 in SDS was comparable to that of NA-CATH in TFE, suggesting that the alterations made in the sequence of NA-CATH:ATRA1-ATRA1 significantly increased its propensity for forming helical structure.

When the peptide sequences are projected on a helical wheel (Figure 4B), the contribution of the substitutions at positions 18 and 25 to a potential hydrophobic face of the NA-CATH:ATRA1-ATRA1 peptide are observed at the top of the helical wheel diagram. On net, the Ala->Phe and Pro->Leu substitutions at positions 18 and 25, respectively, increase the hydrophobicity at those positions, which may improve the interactions between the peptides and the hydrophobic tails in surfactant micelles (and lipid membranes), further stabilizing helical structure in NA-CATH:ATRA1-ATRA1 when interacting with anionic surfactants or lipids. Similarly, if the ATRA2 and ATRA1 peptides are projected individually in helical wheel format, the contribution of these two positions can be seen to the potential hydrophobic peptide face of each peptide (Figure 4C). ATRA-1 may present a more helical face that is also significantly more uniform than that of ATRA-2, with the side chain of phenylalanine at the 3^rd ^position of ATRA-1 exhibiting significantly greater hydrophobic character than the alanine residue at the same position in ATRA-2.

## Discussion

In this study, we tested the *in vitro *susceptibility of *Staphylococcus aureus *to an elapid snake-derived cathelicidin, NA-CATH, as well as related novel, synthetic peptides and compared the performance of these peptides to that of the human cathelicidin LL-37. We demonstrated that LL-37 has similar potency *in vitro *against *S. aureus *to NA-CATH, as opposed to our earlier findings for *E. coli *and other gram-negative bacteria where we determined NA-CATH to be more potent than LL-37 [25,26]. The EC50 values were converted from μg/ml to μM to reflect the number of molecules of peptide and to accommodate the different molecular weights of the peptides. Therefore, on a molar basis, LL-37 was slightly (2.4-fold) more effective against *S. aureus *than the NA-CATH, but the difference was not statistically significant. The EC50 for the D-enantiomer, D-LL-37, was found to be ~10 fold higher than for LL-37, suggesting that it is less effective as an antimicrobial peptide under these conditions for *S. aureus*.

Three 11-residue peptides based on the ATRA motifs of the NA-CATH sequence (ATRA-1, ATRA-2, and ATRA-1A) were compared. The three ATRA peptides all had a nominal charge of +8 at pH 7, and their sequences differed only by the residues at the 3rd (F/A) and 10th position (L/P). On a molar basis, ATRA-1 is significantly more potent against *S. aureus *than ATRA-2, by ~10-fold. We demonstrated in prior work that the presence of alanine and proline at the 3^rd ^and 10^th ^positions, respectively, in the sequence of ATRA-2 (KR**A**KKFFKK**P**K) resulted in a significant alteration to the predicted hydrophobic face of the peptide and disrupted peptide helicity [29]. The peptide ATRA-1A (KR**A**KKFFKK**L**K) was synthesized as a variation on the ATRA-1 peptide sequence (KR**F**KKFFKK**L**K) in order to determine the degree to which the Ala->Phe substitution at the 3^rd ^position contributed to the reduced potency ATRA-2 exhibited against *S. aureus*. ATRA-1A is ~25 times more effective against *S. aureus *than is ATRA-2. However, comparing ATRA-1A to ATRA-1, the alanine substitution did not statistically change its activity against the gram-positive *S. aureus *(1.4 fold, p > 0.05), in contrast to the significantly improved activity against gram-negative bacteria [29]. The side chain of alanine is smaller than phenylalanine, which could affect the peptide's hydrophobic face. The proline residue tends to make the peptide structure destabilized and disrupts the helical structure of peptides. This may impact the ability of the ATRA-2 to achieve a stable and well-defined helical conformation when interacting with bacterial membranes. We conclude that the substitution of alanine in ATRA-1A does not significantly contribute to the antimicrobial activity of the ATRA motif against *S. aureus*. Thus, the presence of the proline residue is likely to be the major contributor to the decreased anti-microbial activity of ATRA-2 peptide [29], and potentially also contributing to the overall anti-microbial activity of NA-CATH.

In earlier work, we demonstrated that ATRA-1 exhibited significant helical character in 60 mM SDS, while ATRA-2 showed no substantial helical character under these conditions. This behavior parallels their anti-microbial potencies. In this study, we found that NA-CATH:ATRA1-ATRA1 had significantly greater helical character in both 50% TFE and 60 mM SDS than did wild-type NA-CATH. In fact, the CD spectrum for NA-CATH:ATRA1-ATRA1 in 60 mM SDS suggests that the peptide has greater helical character under these conditions than the parental NA-CATH does in 50% TFE, a strongly helix-promoting environment. The anionic SDS is frequently used as a model system in studying the interaction between CAMPs and bacterial membranes [36,37]. Accordingly, the increased helical nature/propensity of NA-CATH:ATRA1-ATRA1 could be a significant factor in its ~6 times (p < 0.05) greater anti-microbial potency against *S. aureus *than the parental NA-CATH. Accordingly, the increased helical nature/propensity of NA-CATH:ATRA1-ATRA1 could be a significant factor in its ~6 fold (p < 0.05) greater anti-microbial potency against *S. aureus *relative to the parental NA-CATH.

The range of effective concentrations displayed by these novel AMPs against *S. aureus *varied from 0.51 to 2.85 μg/ml (excluding peptides that proved ineffective). At these concentrations, these peptides have shown no significant hemolytic activity against erythrocytes, implying that the peptides can specifically target the cell membranes of bacteria at their effective EC50 without lysing mammalian cells.

In addition to assessing their anti-microbial activities, the capabilities of the peptides to inhibit *S. aureus *biofilm formation were tested. Biofilm formation by *S. aureus *is clinically relevant because biofilm formation allows pathogens to adhere to and accumulate on scabs or in-dwelling medical devices, such as catheters. Furthermore, in addressing wound infections, biofilm-embedded bacteria are often more difficult to combat than bacteria in planktonic form. This difficulty applies to both antibiotic regimes and the host immune response [38,39]. Thus, it would be beneficial to prevent biofilm production as part of wound treatment. NA-CATH:ATRA1-ATRA1 proved effective at inhibiting biofilm formation at concentrations much lower than is required to reduce bacterial growth under high salt conditions. These findings are important, as there are few reports of AMPs or other antimicrobials exerting anti-biofilm activity against *S. aureus *at sub-anti-microbial concentrations. This suggests that these peptides may act internally on the bacteria, affecting the expression of genes that are essential for the development of biofilm [15,32]. For example, in *S. aureus*, production of PNAG polysaccharide, which is a major component of the biofilm matrix, is regulated by genes of the *agr *locus [40] (in response to an autoinducer peptide, AIP) and the *ica *locus [41]. In addition, a critical role for Bap (biofilm-associated protein) has been demonstrated for biofilm formation by this bacterium, with Bap and genomic DNA (or eDNA) contributing to the strength of the biofilm. In *Pseudomonas aeruginosa*, the human cathelicidin LL-37 alters the expression of biofilm related genes such as Type IV pili, Rhamnolipid and Las quorum sensing system at sub-antimicrobial levels [32]. *Staphylococcus aureus *lacks these genes, and the molecular and genetic targets of LL-37 against *S. aureus *remain undefined.

By performing biofilm attachment experiments against *S. aureus*, we were able to determine that NA-CATH:ATRA1-ATRA1 and its parent peptide, NA-CATH, inhibit biofilm but not by inhibiting attachment. D- and L-LL-37 peptides are capable of inhibiting initial biofilm attachment (58-62%), suggesting a potential interaction of these peptides with bacterial adhesins may be part of their mechanism.

We have not yet determined the bacterial target of NA-CATH:ATRA1-ATRA1 or the D- and L-LL-37 peptides in *S. aureus*, but we intend to investigate this further in future work. One mechanism could be by directly promoting biofilm dispersal (as has been observed for some cationic detergents such as cetylpyridinium chloride [42]) or by inhibiting attachment. It is unlikely that the mechanism involves killing the bacteria, since we have observed that bacterial growth under high-salt conditions is not affected by these peptides. Moreover, anti-biofilm activity was observed for peptides associated with poor anti-microbial effect such as D-LL-37. In addition, we have shown specificity of response, as the scrambled LL-37 peptide does not inhibit biofilm production nor attachment in *S. aureus*.

The *in vivo *relevance of the host cathelicidin response to *S. aureus *infection is not fully established. It has been demonstrated that exposing keratinocytes to live *S. aureus *induces production of beta-defensin peptides, hBD1 and 3, but does not induce expression of hBD2 or LL-37. In addition, intracellular *S. aureus *did not induce LL-37 expression. However, heat-killed *S. aureus *or lipotechoic acid (LTA), a component of *S. aureus *cell wall, were able to induce LL-37 expression in keratinocytes [1]. These studies indicate that the presence of this bacterium in or on the human host may induce the expression of LL-37 *in vivo *under the appropriate circumstances. Finally, in addition to direct effects on the bacteria, these peptides can also exert direct effects on host cells (although they do not appear to lyse host cells at these concentrations). LL-37 may have wound-healing properties [43]. The host targets of LL-37 in human cells were found to include GAPDH [44], EGFR [45,46] and the P2X7 receptor [47]. D-LL-37 has been reported to exhibit powerful immuno-stimulatory activity on the host (more effectively than the L-peptide), such as the induction of IL-8 in keratinocytes and promoting fibroblast proliferation [28], which suggests that it could promote wound healing as an added effect. The bacterial and host-cell targets of these peptides will be the focus of our continued studies.

## Conclusions

Novel treatments for chronic wound infections are critically needed. These wound infections are characterized by the presence of a polymicrobial population of biofilm-forming bacteria, including *S. aureus*. The desired characteristics of a novel therapeutic for treating these wounds would include incorporating the peptides in broad-spectrum, anti-biofilm, topical treatments with wound-healing properties. In this work, we examined the anti-biofilm activity of two synthetic cathelicidin-like synthetic peptides against *S. aureus*.

Overall, our results suggest that novel synthetic peptides can be designed based on naturally occurring cathelicidins, peptides which demonstrate similar or improved potencies relative to that of the parent full-length AMPs. Exemplifying this proposition, the highly-effective anti-microbial peptide NA-CATH:ATRA1-ATRA1 not only displayed improved anti-biofilm activity relative to parent peptide, but it also exhibited enhanced anti-microbial activity. D-LL-37 represents a protease-resistant peptide mimetic that was as effective as the L-peptide isomer LL-37 at inhibiting biofilm formation. Furthermore, D-LL-37 may possesses wound-healing properties towards the host.

These peptides may have potential to be developed as topical treatments against infections involving biofilm-forming bacteria, such as *S. aureus*, reflecting the modern understanding of the role of biofilms in chronic wound infections. It may be important to consider these peptides as anti-biofilm perhaps more so than anti-microbial, reflecting their capabilities under *in vivo *salt conditions. We have demonstrated that these peptides exert broad-spectrum activity against both gram-positive and gram-negative bacteria, and thus could be useful in the treatment of patients with polymicrobial wounds infections [6,7].

## Methods

### 5.1 Bacterial strains and media

*S. aureus *(ATCC 25923, American Type Culture Collection, Manassas, VA) was grown in Nutrient Broth (Difco Laboratories, Detroit, Mich.) at pH 7, 37°C, 24 h with shaking at 200 rpm. The overnight culture was frozen with 20% glycerol and stored at -80°C. The frozen stock was enumerated (CFU/ml) by dilution plating and growth on Nutrient Agar plates.

### 5.2 Peptides and Anti-microbial assays

The sequences and net charges of the peptides are shown in Table 1. The molecular weight reported here for each peptide reflects the trifluoroacetic acid (TFA) salt form of the peptides. NA-CATH, NA-CATH:ATRA1-ATRA1, ATRA-1, ATRA-1A, ATRA-2 peptides (86.1 and 89.7, 97.2, 94.5, and 88.2%, respectively) (Genscript, Piscataway, NJ), LL-37 (95% purity) (AnaSpec 61302) and D-LL-37 (92.0% purity) (Lifetein, South Plainfield, NJ) were synthesized commercially.

The anti-microbial activity of the NA-CATH and NA-CATH:ATRA1-ATRA1, the variations on the ATRA peptides LL-37 and D-LL-37 against *S. aureus *were determined as previously described, with some modification [26,29]. For anti-microbial assays, frozen enumerated aliquots were thawed and gently mixed immediately before use. In a 96-well plate (BD Falcon 353072), 1 × 105 CFU per well bacteria were incubated with different peptide concentrations (in serial dilutions of 1:10 across the plate) in a solution of buffer containing sterile 10 mM sodium phosphate (pH 7.4) and incubated (3 h, 37°C). Negative control wells contained bacteria with no peptide. Serial dilutions were then carried out in sterile 1x PBS (Fisher Scientific) (pH 7) and plated in triplicate on Nutrient Agar plates, incubated (37°C, 24 h) and counted. Bacterial survival at each peptide concentration was calculated as previously described [25,26] based on the percentage of colonies in each experimental plate relative to the average number of colonies observed for assay cultures lacking peptide. The EC50 was calculated as previously described [26,47].

Each experiment was repeated at least twice, and a representative experiment is shown, for clarity. Errors were reported based on the standard deviation from the mean of the log10 EC50 values [19]. 95% confidence intervals were used to determine whether points were statistically different at p = 0.05.

### 5.3 CD Spectroscopy

Circular dichroism (CD) spectra of the peptides were collected using Jasco J-815 spectropolarimeter. Samples were allowed to equilibrate (10 min, 25°C) prior to data collection in a 0.1 cm path length cuvette, with a chamber temperature 25°C throughout each scan. Spectra were collected from 190 to 260 nm using 0.2-nm intervals; 3 scans per sample were averaged. All peptides were analyzed at 250 μg/ml concentration in multiple mediums: 10 mM sodium phosphate (pH 7), 50% (v/v) trifluoroethanol (TFE) in 10 mM sodium phosphate (pH 7), and 60 mM sodium dodecyl sulfate (SDS) in 10 mM sodium phosphate (pH 7) [34]. Helical wheel projections were performed as described in the figure legend (Figure 4B, C).

### 5.4 Biofilm production

Biofilm production was measured as previously described [48] with the following modifications. *S. aureus *(1 × 10^5 ^CFU) in 200 μl of sterile trypticase soy broth media (TSB) (Becton, Dickinson and Company) (pH 7) was incubated with either with no peptide, NA-CATH:ATRA1-ATRA1, NA-CATH, LL-37, D-LL-37, or scrambled LL-37 at concentrations of 1.0, 0.1, and 0.01 μg/ml (24 h, 37°C) in a 96 well plate (BD Falcon 353072). The positive control is *S. aureus *in TSB with no peptide. Six wells were used for each peptide concentration (n = 6). After 24 h, the optical densities (OD) of the wells were taken at 600nm to quantify biofilm formation. The biofilm production was measured using the crystal violet stain technique [48].

All experiments were repeated at least twice, with a representative experiment shown.

### 5.5 Biofilm attachment assay

Biofilm attachment assays were performed in a 96-well microtiter plate (BD Falcon 353072), as previously described [32]. Overnight cultures of *S. aureus *were grown in TSB to an optical density (600nm) of ~1.0. 200 μl culture was added to the wells, followed by no peptide, scrambled LL-37 , LL-37, D-LL-37, NA-CATH, or NA-CATH:ATRA1-ATRA1 at 1 μg/ml. The plates were incubated (1 h, 37°C) for *S. aureus *to adhere to the wells. The wells were washed and OD_600 _measurements were taken, as in the biofilm production experiments, and the average absorbance for each treatment was determined (n = 16).

### 5.6 Hemolysis assay

Hemolytic activities of the peptides were determined using equine erythrocytes (Hema Resource Inc., Eugene, OR, USA) in an assay adapted to a microtiter plate format [29]. Briefly, erythrocytes were prepared by centrifuging 1 ml fresh defibrinated blood (1620 × g, 10 min), re-suspending the pelletted cells in 1 ml sterile PBS (Fisher Scientific) (pH 7). The cells were washed with PBS three times; in the final wash the cells were re-suspended in 0.75 ml PBS. From this, a 2% erythrocyte suspension was prepared for the assay. Aliquots of sterile water (positive control), peptide, and PBS (negative control) were used in a microtiter plate. Various peptide concentrations in sterile 10 mM sodium phosphate (0.1, 1, 10, 100 μg/ml) were tested in n = 12. The assay was then incubated (1 h, 37°C). After centrifugation (1000 × g, 10 min), aliquots of supernatant were carefully transferred to a new microtiter plate and the absorbance was obtained for each well. Percent hemolysis was calculated as previously described [26].

### 5.7 Statistical Analysis

Antimicrobial assay measurements were performed in triplicates, biofilm assays with n = 6. Standard deviations of the mean of each set are represented on each graph. Where the error bars cannot be seen, the error is very small. Confidence Interval (CI) (95%) is presented demonstrating the statistical overlap of the data. For all other assays, p-values were determined by performing a standard T-test.

## Authors' contributions

SD carried out the anti-microbial, hemolytic, and biofilm assays, analyzed the data and contributed to writing the manuscript. BB designed the peptides and carried out the circular dichroism experiment, interpreted the results, and contributed to writing the manuscript. MVH conceived of the overall study, designed and coordinated the experiments, and wrote the manuscript. All authors read and approved the final manuscript.
